# Current state of professional and core competency in pediatric residency program at Shiraz University of Medical Sciences: A local survey

**Published:** 2015-10

**Authors:** SEDIGHEH EBRAHIMI, RITA REZAEE

**Affiliations:** 1 Medical Ethics Department, Medical School, Shiraz University of Medical Sciences, Shiraz, Iran;; 2 Quality Improvement in Clinical Education Research Center, Shiraz University of Medical Sciences, Shiraz, Iran

**Keywords:** Accreditation, Standard, Pediatric

## Abstract

**Introduction:**

Accreditation assesses performance, or capacity to perform, against predetermined standards. It typically combines external quality assurance, through a process of peers review, with elements of self-regulation through internal and self-directed assessment. This study is an attempt to identify the quality of pediatrics residency educational programs regarding predetermined standards.

**Methods:**

This descriptive-analytical evaluation study of applied type was conducted during 2010 and 2011 in the pediatrics department of Shiraz Medical School, Iran. The assessment process occurred in several phases; at first an assessment model for a residency educational development and a series of educational criteria and indices were created based on WFME Standards. Multiple methods including a self-assessment questionnaire and several checklists were used to collect data, whereas systematic site visit, peer review and document reviewing were conducted with survey team. Due to limitation of the statistical society, all faculty members (n=34) and residents (n=41) of the pediatric department were asked to complete the survey. At last, descriptive and deductive statistics data analysis was performed using SPSS version 14.

**Results:**

According to the records available in assessing program quality, it seems that the input criteria were desirable for the program based on the residents’ viewpoints (86.6 %).There were proper physical facilities for them to meet the residency program goals.  The study indicated that the learning environment needed to be revised for the educational needs (Likert scale: 2.96±1.05). The peer evaluation team demonstrated achievement of mission fulfillment in the context of the objectives and indicators by meeting the desired themes. * *In spite of some weaknesses in the process criteria, the criteria for output indicators were good according to the report (more than desired level of 75-80%).

**Conclusion:**

Accreditation is an important step towards strengthening the quality of educational programs. According to this study the current status of the pediatrics department of Shiraz University of Medical Sciences was desirable leading to a satisfactory level in general. However, additional educational development will be needed in order to achieve a widespread change and improvement.

## Introduction

The residency is a structured educational program that is expected to provide acceptable performance to all enrolled residents. The World Federation for Medical Education (WFME) defines the educational standards to evaluate postgraduate medical students during their education (1).

In recent years, policy-makers and educators in the Islamic Republic of Iran, have expressed their concerns about the quality of undergraduate (MD degree) and postgraduate medical education. In response to this raising concern, they have focused on quality improvement and have paid greater attention to accreditation as a tool for evaluating the quality of educational programs in medical schools (1).

In order to develop efficient assessment measures, they have moved toward an emphasis on central supervision on the quality of education and competency programs for residents. To achieve this goal, Iranian Council for Graduate Medical Education (ICGME) was established by the national parliament in the early 1970’s. One of the tasks of the Council was to supervise specialty education units in medical schools around the country. For this purpose, the Accreditation Commission and Specialty Review Committees were set up under the ICGME scheme (1, 2).

Furthermore, a great effort was made to implement a government-mandated accreditation system and as a result, some educational guidelines for assessment of medical trainees were developed.  

*Like many of Iran’s medical universities*, in 2010, Dean of Shiraz University of Medical Sciences considered the investment of time to promote the quality of residency educational programs. The first step in this way was the establishment of the accreditation committee to provide the highest educational standards in all fields of specialty regarding the previously performed internal evaluation (self-study). The Committee had responsibility to execute the external evaluation of the residency programs before commencing the summative evaluation and ultimately determination of their accreditation status.

To ensure medical residents’ competencies, transparent documentation of the quality levels of educational programs was essential.

In accordance with this mandate*, *Educational Development Center (EDC) appointed a Task Force to *work* on milestones for medical residency training. In the early stages of the process, EDC and the department of pediatrics collaborated to initiate the pediatrics milestones project.

The first *refined* set of educational standards in pediatrics specialty, based on WFME Standards were determined and localized in accordance with the Iranian Council for Graduate Medical Education.  After achieving the support of university chancellor, the chair and residency program director of pediatrics department were informed about the assessment system (1, 3).

 In this article, we review what was done in a descriptive evaluation study using of combination of self-review and peer evaluation through the pediatrics residency accreditation process. 

It indicates an analysis of the strengths and weaknesses of the implementation of standards and the *factors* influencing the preparation of residents for clinical practice, focusing on the *assessment* of the educational programs *quality *of the pediatrics department in Shiraz Medical School, Iran. There is an attempt to identify departmental performance regarding input, process and output standards areas. As such, it reviews the eligibility requirements, mission, and extent of mission fulfillment, and core themes with objectives and indicators of achievement. This paper is intended to clarify the need for fresh approaches to the residency educational programs for further development of contemporary and future medical practice. It also describes responsibilities of EDC regarding the quality of medical education.

## Methods

This descriptive-analytical evaluation study of applied type was conducted during 2010 and 2011 in the pediatrics department of Shiraz Medical School, Iran. The accreditation of medical programs is carried out by survey teams including medical educators and pediatrics practitioners, appointed by the Vice-chancellor for Education. Due to limitation of the statistical society, all faculty members (n=34) and residents (n=41) of pediatric department were asked to complete the survey. The assessment process occurred in several phases of planning, complementary and analysis. 

At the first step, after appropriate planning, EDC Project Working Group searched the medical literature to find an assessment model for a residency educational development and a series of educational criteria based on WFME Standards were created. A group of academics and clinical* experts* gave their opinion about standards based on "importance" and "feasibility", using two rounds Delphi’s technique. They were asked to vote the standards, engaging in an iterative process of revisions until reaching consensus.

After determining the standard domain of the department, the baseline data already provided by the internal evaluation were reviewed and measurable indicators, evidence and grading were developed. Then appropriate methods and instruments for proper data gathering were designed and checked for validity and reliability. The tools for data collection included multiple methods such as a self-assessment questionnaire, several information checklists, systematic site visits, document reviewing, interview and observation forms. 

In the second step a team of medical educators and practitioners visited the pediatrics wards and evaluated the program against already developed criteria. The measurement of each standard was carried out by reviewing documents and statistics; individual interviews (with the department head and program director) and group interviews (with the residents and students, etc.); and by direct observation (at visits to facilities, departments and outpatient clinics). During the data gathering stage, the necessary information about the pediatrics department to complement the internal evaluation and database information was added. The educational program was evaluated from the residents’ points of view using  a reliable and valid self-administered questionnaire with 17 items and the format of a five-level Likert-type scale )1 for ‘not at all important’ and 5 for ‘very important’).

The content validity of the questionnaire was evaluated through expert judgments and its reliability and internal consistency were tested using test-retest reliability with correlation coefficient of 0.84. The residents’ performance assessment (the core skills) was done using different methods such as CBD (Case Based Discussion), OSCE (Objective Structure Clinical Examination) one time per year, Mini CEX (monthly), DOPS (Direct Observation of Procedural Skills), Logbook (monthly) and MCQ (two times per year).

In the third step the collected data were coded and analyzed through descriptive and deductive statistics using SPSS, version 14. The team recorded their analysis of the strengths and weaknesses of the findings and documents. They delivered a report on evaluation about teaching plan and curricula, educational resources and facilities, the teaching staff members and relevant residents’ information. Also, the report included educational strengths and weaknesses, related performance problems that require attention and distinctive activities, points of non-compliance with the accreditation standards and areas in which changes are foreseen, such as those with an expectation to improve. 

 Then the first report was sent to the department of pediatrics and called for their interpretation in two focus group sessions. Also the pediatric faculties and leaders were informed about the planned site visit. They were given the opportunity to design strategies to ensure successful problem resolution. 

In next step with the agreement of the department and university, in the management of the assessment process, another department of pediatrics in the country was appointed to come to Shiraz as peer reviewers and external evaluators. The purpose of the site visit was to provide an external validation of the results of the self-evaluation regarding fulfillment of the standards and, if necessary, to acquire supplementary information. 

 On October 2011 the external evaluator team came and visited all parts of the educational program in this department including all educational wards in the teaching hospitals in Shiraz. The duration of site visits was two days. Immediately after the visit, the external evaluators summarized their preliminary findings and presented them in a private meeting with the dean. Then, the team ended their review with feedback to the pediatrics department. Furthermore, they submitted their written draft report, including the recommendations, in order to correct any errors. 

## Results

The present study indicated that there were 34 teachers (attending physicians) supervising 41 residents (a ratio of 34/41). It means there was almost one teacher for each resident (Desired: one teacher for every 3 residents). More than 70% of teachers were associate professors and full professors in pediatrics, which was desirable.

The survey showed there were one resident and one nurse for every 4 patients. Ideally, there should be at least one nurse for every 3 pediatric patients. *Beds Occupancy* Rate was 83.8% (desired more than 75%).

Access to appropriate learning opportunity was expressed by 86.6% of residents.

The residents’ points of view about the program‘s evidence are shown in table 1. In this part a researcher- made questionnaire was used based on five-level Likert-type scale with a mean ≥3 as desired, 2 £ mean £3 as for the need to revise and mean £ 2 as not desired. Content and face validity was evaluated by the opinions of the experts. The reliability of the questionnaire was found to be 0.84, using Cronbach’s Alpha.

**Table 1:** Evidence demonstration of the program from residents’ points of view

**Items**	**Mean±SD**
**Availability of teachers**	3.13±1.05
**Learning environment**	2.96±1.05
**Supervision and feedback**	2.63±1.05
**Clarity of task analysis**	2.8±1.2
**Education programs**	3.38±1.1
**Enough time to self-assessment**	1.40±0.82

Sixty seven percent of the residents stated that they had experienced inadequate on-site faculty presence and supervision, whereas 24.6% of them said that they had felt adequate on-site faculty presence and supervision and the remainder (8.4%) said they had experienced excessive supervision.

This document also provided an overview on the intended objectives of the educational programs. It covered a number of issues regarding the overall experiences and activities of the residents as shown in the following results. 

The residents reported that on average, there were 5 attending rounds per week, with teaching sessions lasting approximately 3 hours. While good availability of teachers was reported by 53.3 % of the respondents, 54% of them believed that the magnitude of teachers’ role in learning processes faded into insignificance. The residents’ participation in ambulatory care rotations was thirteen percent of the entire outpatient track.

In addition to the regular clinical learning practices, the supplementary workshops are included in the residents’ training programs. These workshops feature sessions on an array of topics including teaching methods, medical ethics in pediatrics, research methodology, communication and managerial skills, introduction to evidence based medicine and family consulting. Full attendance at the workshops is mandatory for residents.

The desired indicators and the current situation of the educational activities are *shown* in table 2.

**Table 2:** The desired indicators and the current situation of the educational activities

**Current situation**	**Desired indicators**	**Activities**
** ✔**	5 days/ week 3 hours/day	Teaching round
**✔**	Every day	Resident teaching round
**Once/month in General wards once/week in subspecialties**	Once/two month	Grand round
**2 days/week in general wards every day in subspecialties**	2-3days/week	Morning report
**Once/month**	Twice/month	Radiology round
**✔**	Once/week	Attending conference
**Once/ week**	1-2/week	Resident conference
**✔**	Once/week	Interdisciplinary conference
**Once/week in subspecialties**	Twice/month	Journal club
**Once/month in general wards** **Once/week in subspecialties**	Once/week	Mortality and morbidity conference

Regarding time allocation, the residents reported sufficient time spending in formal pediatric related conferences (60%) with a mean of 6 hours per week. Fifty eight percent of the residents thought that the planned training activities were appropriate, 13.3% believed that those activities were too many and 28.7% believed that those activities were not enough.

Additional findings about the performance of the residents by self assessment are indicated in table 3.

**Table 3:** Results of residents’ self-assessment

**Items**	**Very good**	**Acceptable**	**Poor**
**Ability in clinical skills**	26.7%	66.7%	6.7%
**Ability to communicate with child and family**	26.7%	73.3%	-
**Communication with other professionals**	20%	53.3%	26.7%
**Ability to self improve and use learning opportunities**	13.3%	40%	46.7%

The pediatric board pass rate over the past three years has been 87% (desired more than 75%), the percentage of success in pediatric board certifying exam and  the pass rate in yearly promotion examination 100% (desired more than 80%). The residents have had the highest board certifying exam score among the residents in the country over the past years. 

## Discussion

In 2010, the Dean of the Shiraz University of Medical Sciences decided to establish an Accreditation Committee to provide guidance in all specialties. According to one of the EDC responsibilities and authorization in providing an ongoing assessment of medical education programs, the Vice-chancellor for Education obliged the EDC to evaluate the teaching programs of medical specialties. The purpose of this assessment process was improvement of residency training and recommendation of the required and appropriate changes. 

The mission and objectives of the Program Evaluation were first defined by the EDC team at that time.

In this study a booklet describing the established assessable standard criteria was developed and given to the teaching staff of the pediatric ward. It covered the qualification criteria for resident training. Each of the assessment areas was subdivided into more detailed as input, process and output criteria. The input criteria included the teachers and their qualifications, the residents, the support policies, facilities, and fiscal and physical resources devoted to the program. The process criteria were defined as resident activities for acquiring good competency, such as teaching programs, bedside teaching and conferences. Output criteria showed a certain level of competency that residents should achieve, for example, performance on examinations, performance in the internal evaluation related to educational program objectives, ranking in national board exam (4), and residents’ satisfaction with the program. All educators agreed on these criteria for residents.

Following the model prepared by the Accreditation Committee, pediatrics’ residency educational programs were assessed for their compliance with the standards.

In the face of input criteria, according to the records available for external verification, it seems that the input criteria were desirable for the program. Those documents showed that over the past three years the department has experienced significant growth in the number of full-time staff dedicated to teaching. Work and teaching rounds are conducted daily by full-time teaching staff. The diversified interests of the full-time staff enhance the resident’s learning experience.

The learning environment including training facilities and various teaching opportunities will enable the residents to promote the competencies and skills needed to provide an up-to-date and broad educational experience to practice excellent general pediatrics in the community (5).The learning environment must be adequate for the physical, psychological and educational needs of all residents and be conducive to proper education of the residents in the competency areas (6, 7). The present study indicated that the residents engaged in learning when using the most current technological gadgets. Also there were appropriate physical facilities provided for them to meet the residency program goals. This includes access to appropriate food services, adequate classroom spaces as well as adequate on-call rooms. In this study, the number of admissions and high rates of Beds* Occupancy* Rate and daily variations in bed occupancy and a variety of patients admitted in the teaching hospitals, indicated efficient resource for learning practices. This is, of course, an over-simplification because these ratios are not a reflection of the availability of new patients, so it is better to look at patients’ turnover intervals not just *Beds Occupancy* Rate (8).

As indicated in table 1, in assessing the program quality, considering residents’ evaluation of their teachers as well as learning environment, they were asked if the training programs and the opportunities were fit for the purposes. The findings revealed that the number, availability of the teachers and the residents’ satisfaction with the quality of attending doctors for the program were desirable.

 The residents should be encouraged to evaluate the quality of their own activities and employ self-correction. 

However, due to 80 hours of mandatory work per week, there was not enough time for self-assessment as they have reported (1.4±0.8). There has been concern that residents were unhappy with learning environment, inadequate supervision and feedback and clarity of task analysis. The inadequacy of supervision may lead to the negative residents’ experiences and behaviors. It may be a sign of a breakdown in the residency educational programs. The current situations of the educational activities match the desired indicators which help the working team if they have met the aimed standards. 

The peer evaluation team demonstrated achievement of *mission fulfillment* in the context of the objectives and indicators by meeting the desired themes. The rresidents’ participation in journal club, case presentations, and seminar were desirable.

The data about the output of the educational program represents an indicator of quality of residents' training, concerning the vision, mission and objectives of the department. It also provides the information concerning the residents’ satisfaction, the percentages of their success in different study materials, their study levels and the pass-rate in the medical residency board examination and pediatrics ward’s academic degrees as some criteria for output indicators, which were good according to the report. Job satisfaction for the teaching staff was not evaluated.

The results of the performance grading of the residents through self-assessment guides the working team and instructor to decide whether the performance was very good, acceptable or poor and insufficient. The findings of this survey indicated that capacity for self-improvement and use of learning opportunities for self-learning were poor (46.7%) to acceptable (40%).

Overall, these are suitable elements for residency training which could not be obtained through didactic medical education. Residents are expected to demonstrate interprofessional collaboration and communication skills that result in positive outcomes such as improved exchange of information, more effective interventions, and promotion of collaboration between health professionals and increased satisfaction of patients and their families (9). Outpatient clinical rotations rate were not desirable (<%13) in this study (Desired is more than %40).

The pediatrician should excel in knowledge, skills, and effectiveness in the ambulatory care setting (10).

Considerable emphasis is placed on pediatric outpatient medicine. For this reason, pediatric residents need to be strengthened in ambulatory care practices in order to develop rigorous practice-based residency training programs.

The most recent guidelines of the “Accreditation Council of Graduate Medical Education Guidelines” now state that at least one-third of residents’ training must be completed in the ambulatory care setting (11-13).

The items indicated in residents’ self-assessment point to the need for attention to the quality of training programs. There is a need to introduce more supplementary educational programs and managerial skills including professional and clinical skills, medical ethics in pediatrics, team working and communication skills to the residents. 

## Conclusion

The current status of the pediatrics department of Shiraz University of Medical sciences was desirable in general. However, additional educational development will be needed in order to achieve widespread change and improvement. 

There should be some programs for revising and expanding learning objectives to reflect the required competencies. There is a need to strengthen educational programs by adding learning opportunities for the competencies, and enhancing processes for evaluating residents’ learning and performance feedback. Particularly, multi-source feedback and direct observation should be included and the faculty members should be involved in development sessions in order to enhance their understanding of the competencies and to increase their skill in evaluating resident performance and provide substantive feedback. 
